# Analysis of the finasteride treatment and its withdrawal in the rat hypothalamus and hippocampus at whole-transcriptome level

**DOI:** 10.1007/s40618-024-02345-y

**Published:** 2024-03-17

**Authors:** S. Giatti, L. Cioffi, S. Diviccaro, R. Piazza, R. C. Melcangi

**Affiliations:** 1 Dipartimento di Scienze Farmacologiche e Biomolecolari “Rodolfo Paoletti”, Università degli Studi di Milano, Via Balzaretti 9, 20133 Milan, Italy; 2 Dipartimento di Medicina e Chirurgia, Università di Milano-Bicocca, Milan, Italy

**Keywords:** 5 alpha-reductase, Male rat, Post-finasteride syndrome, Side-effects, RNA sequencing analysis

## Abstract

**Purpose:**

As reported in patients treated for androgenetic alopecia with finasteride (i.e., a blocker of the enzyme 5 alpha-reductase) and in an animal model, side effects affecting sexual, psychiatric, neurological, and physical domains, may occur during the treatment and persist with drug suspension. The etiopathogenesis of these side effects has been poorly explored. Therefore, we performed a genome-wide analysis of finasteride effects in the brain of adult male rat.

**Methods:**

Animals were treated (i.e., for 20 days) with finasteride (1mg/rat/day). 24 h after the last treatment and 1 month after drug suspension, RNA sequencing analysis was performed in hypothalamus and hippocampus. Data were analyzed by differential expression analysis and Gene-Set Enrichment Analyses (GSEA).

**Results:**

Data obtained after finasteride treatment showed that 186 genes (i.e., 171 up- and 15 downregulated) and 19 (i.e., 17 up- and 2 downregulated) were differentially expressed in the hypothalamus and hippocampus, respectively. Differential expression analysis at the drug withdrawal failed to identify dysregulated genes. Several gene-sets were enriched in these brain areas at both time points.

**Conclusion:**

Some of the genes reported to be differentially expressed (i.e., *TTR, DIO2, CLDN1, CLDN2, SLC4A5, KCNE2, CROT, HCRT, MARCKSL1, VGF, IRF2BPL*) and GSEA, suggest a potential link with specific side effects previously observed in patients and in the animal model, such as depression, anxiety, disturbance in memory and attention, and sleep disturbance. These data may provide an important background for future experiments aimed at confirming the pathological role of these genes.

**Supplementary Information:**

The online version contains supplementary material available at 10.1007/s40618-024-02345-y.

## Introduction

Finasteride, a blocker of the 5 alpha-reductase (i.e., the enzyme converting testosterone into dihydrotestosterone and progesterone into dihydroprogesterone) is clinically used for benign prostatic hyperplasia and androgenetic alopecia [1]. Even if the efficacy of this drug is well established in both disorders, several studies have reported important side effects during the treatment, and persistence of them at the drug suspension, with the appearance of the so-called Post-finasteride syndrome (PFS) [1–8]. In particular, PFS patients reported side effects in the sexual domain, such as erectile dysfunction, loss of libido and sexual drive, penile atrophy, and diminished ejaculatory [9–14]. In addition, psychiatric, neurological and physical domains, such as depression, anxiety, panic attacks, reduction in self-confidence, disturbance in memory and attention, sleep disturbance, peripheral neuropathy, genital numbness and paresthesia, muscular atrophy and alteration of fat distribution have been reported [4, 6–8, 12, 13]. To date, the biological basis of these side effects has been poorly explored. Indeed, the observations present in the literature are mainly based on symptoms self-reported by the patients and only a few papers have deeply investigated these aspects. For instance, as demonstrated in PFS patients [13, 15, 16] and in an animal model [17], finasteride treatment is not only able to block the enzyme 5alpha-reductase and consequently the metabolism of testosterone and progesterone, but has a broad consequence on the pattern of several other steroids. Indeed, it is able to affect the plasma and brain levels of neuroactive steroids (i.e., a family of steroids, including steroid hormones and neurosteroids, which affects nervous functions). Interestingly, not only their levels but also alterations in their mechanism of action (i.e., via classical and nonclassical steroid receptors) have been reported [17–20]. Accordingly, the important role of neuroactive steroids in regulating nervous functions [21], human and animal PFS studies have ascertained impaired sexual function, depressive symptomatology and alterations in gut microbiota composition and gut–brain axis [12, 13, 22–25]. In particular, in the animal model, depressive-like behavior was associated with increased hippocampal neuroinflammation, altered neurogenesis, and increased reactive astrogliosis [24]. In addition, finasteride is not only an inhibitor of the 5 alpha-reductase but as recently demonstrated it is also able to block the enzyme phenylethanolamine N-methyltransferase, that it is responsible for the conversion of norepinephrine into epinephrine [26]. Thus, finasteride may alter per se this important neurotransmitter system. Recent observations, obtained in penile skin samples by microarray, have shown that 1.446 genes and 2.318 were overexpressed and underexpressed respectively, in PFS patients vs healthy controls [27], suggesting that gene expression differences may be a potential etiology of side effects occurring in these patients. On this basis, by RNA sequencing analysis, we have here evaluated the effect of finasteride chronic treatment (i.e., for 20 days) and its withdrawal (i.e., for 1 month) in two important brain areas of adult male rats, possibly related to the side effects induced by finasteride, such as the hypothalamus and hippocampus.

## Materials and methods

### Animals and treatments

Adult male Sprague–Dawley rats (200-225 g at arrival, Charles River Laboratories, Italy) were used. All procedures were carried out in the animal care facility of the Department of Pharmacological and Biomolecular Sciences (DiSFeB) at the Università degli Studi di Milano, Italy and were approved by the local ethics committee and the Italian Ministry of Health (authorization 1083/2015-PR). All manipulations were performed in accordance with national (D.L. No. 26, March 4, 2014, G.U. No. 61March 14, 2014) and international laws and policies (EEC Council Directive 2010/63, September 22, 2010: Guide for the Care and Use of Laboratory Animals, United States National Research Council, 2011). Rats (*n* = 24) were acclimated to the new environment for 1 week. Finasteride (1 mg/rat/day; Sigma-Aldrich, Italy) was dissolved in a vehicle solution of sesame oil and ethanol (5% v/v) and administered subcutaneously for 20 days at a volume of 100 μL/day. Finasteride and vehicle-treated rats were sacrificed at 24 h (*n* = 4 for each group) after the last injection and 1 month (*n* = 4 for each group) after drug suspension. After sacrifice, hippocampus and hypothalamus were dissected and immediately stored at − 80 °C until the analysis.

### RNA extraction

Total RNA from the hippocampus and the hypothalamus was extracted using Trizol (Invitrogen, San Giuliano Milanese, Italy). Briefly, tissues were homogenized with the Tissue Lyzer instrument (Qiagen, Milan, Italy), and chloroform was added to obtain phase separation. RNA was present in the upper aqueous phase, and its separation was obtained with a Directzol™ RNA MiniPrep kit (Zymo Research, Irvine, CA, USA) in accordance with the manufacturer’s protocol and as previously reported.

### Whole transcriptome sequencing

Total RNA was quantified by NanoDrop™2000 (ThermoFisher scientific, Milano, Italy) and its integrity was verified with the Agilent TapeStation system (Agilent, Santa Clara, USA). RNA integrity number (RIN) > 7.5 was considered sufficient for further analysis. Then, Illumina stranded mRNA prep (Illumina, San Diego, USA) was used according to the manufacturer’s protocol to prepare libraries that have been sequenced into a NextSeq 550 instrument (Illumina, San Diego, USA).

### Data processing and bioinformatics analysis

Raw sequences were initially tested using FastQC (https://www.bioinformatics.babraham.ac.uk/projects/fastqc/). Subsequently, fastq reads were aligned against the reference Rattus Norvegicus genome using the splice-aware aligner Star [28], using the quantMode GeneCounts parameter to perform raw counting at gene level. The Bioconductor package DESeq2 v. 1.30 [29] was applied to perform the differential gene expression analyses. Differential genes were identified by selecting a Benjamini–Hochberg adjusted *p*-value < 0.1. Bam alignment files were indexed using Samtools [30] generating the bam-associated bai index files. The sorted, indexed bam alignment files, together with bai indexes, were then manually inspected using the Integrative Genomics Viewer [31]. GSEA were carried out using the GSEA tool v. 4.2.1 (https://www.gsea-msigdb.org/gsea/downloads.jsp) by applying 1000 permutations at gene_set level. Gene-sets with a Benjamini–Hochberg adjusted *p* value < 0.25 were considered statistically significant.

## Results

**Fig. 1:**
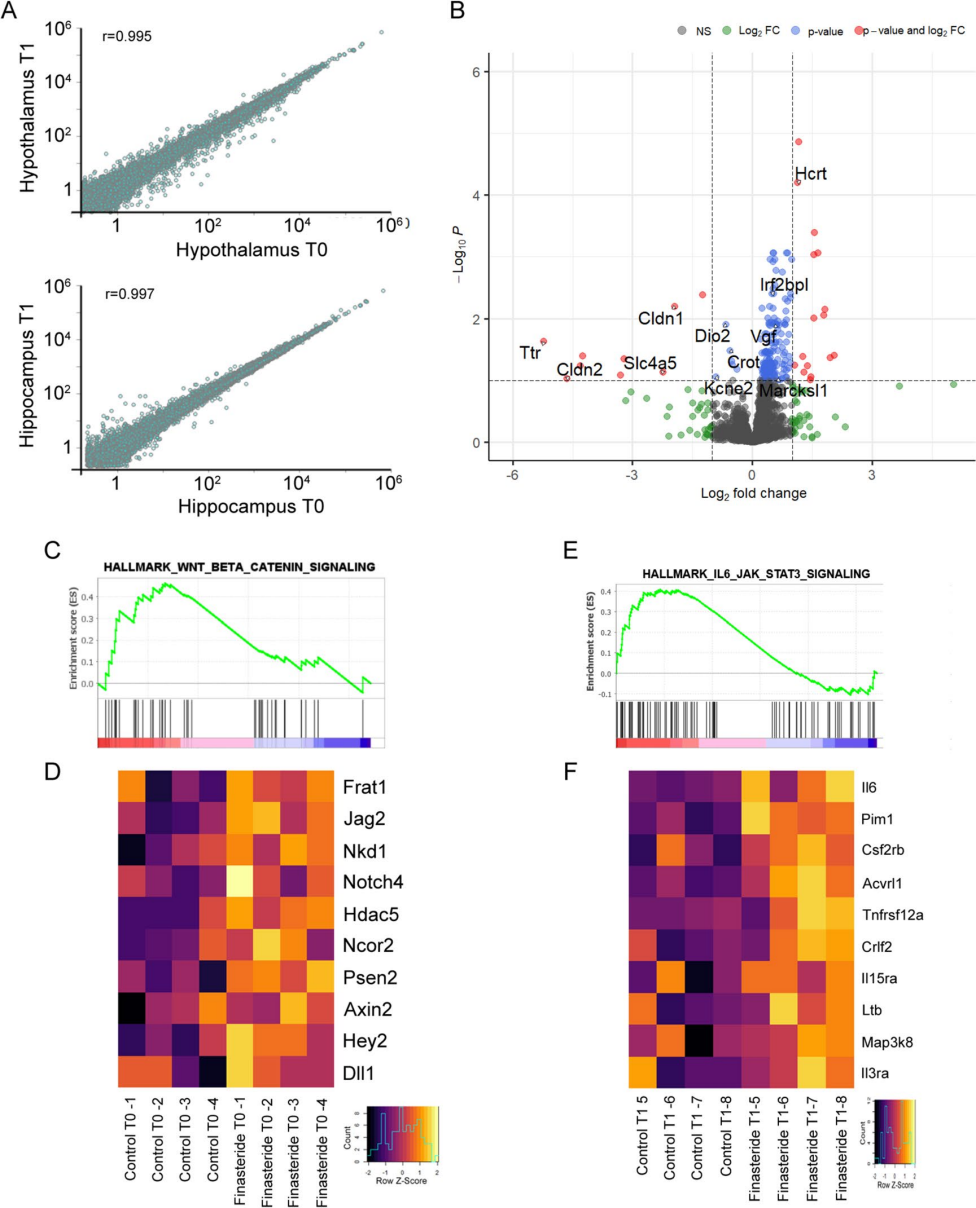
**A** Pearson correlation analysis of whole-transcriptome case/control Log_2_-FoldChange ratios in Hypothalamus (upper panel) at T0 (*x* axis) vs T1 (*y* axis) and in Hippocampus (lower panel) at T0 (*x* axis) vs T1 (*y* axis). **B** Volcano plot showing the whole-transcriptome case/control Log_2_-FoldChange ratios (*x* axis) and the associated Colog_10_ transformed *p* value in Hypothalamus at T0. Grey dots highlight genes non-significant and with absolute Log_2_-FoldChange ≤ 1; green dots genes with absolute Log_2_-FoldChange > 1 and -Log10 *p*-value < 1; blue dots genes with absolute Log_2_-FoldChange < 1 and -Log10 *p*-value > 1; red dots genes with absolute Log_2_-FoldChange > 1 and -Log10 *p*-value > 1. **C** GSEA plot of the WNT-beta-catenin and **D** associated heatmap in Hypothalamus at T0 in control and Finasteride-treated rats. **E** GSEA plot of the IL6-JAK-STAT3 signaling and **F** associated heatmap in Hypothalamus at T1 in control and Finasteride-treated rats.*n* = 4 for each experimental group

A correlation analysis done at whole-transcriptome level in rat hypothalamus and hippocampus at the two time points in presence vs absence of finasteride showed a very strong correlation for hypothalamus treated or not treated with finasteride after chronic treatment (T0) or at withdrawal (T1) (Pearson’s *r* = 0.995) as well as for hippocampus at T0 vs T1 (Pearson’s *r* = 0.997), suggesting a similar transcriptional effect of finasteride at the two different time points (Fig. 1A).

To isolate the transcriptional programs associated with finasteride treatment in the hypothalamus at T0, we initially performed a differential expression analysis, which revealed 186 differentially expressed genes. Among these, 171 and 15 genes were up- and downregulated, respectively (Supplementary Table 1). In particular, we reported altered genes, such as Transthyretin (*TTR*), Iodothyronine Deiodinase 2 (*DIO2*), Claudin 2 (*CLDN2*) and 1 (*CLDN1*), Solute Carrier Family 4 Member 5 (*SLC4A5*), Potassium Voltage-Gated Channel Subfamily E Regulatory Subunit 2 (*KCNE2*), carnitine octanoyltransferase (*CROT*), Hypocretin Neuropeptide Precursor (*HCRT*), myristoylated alanin-rich C-kinase (*MARCKSL1*), Interferon Regulatory Factor 2 Binding Protein Like (*IRF2BPL*)*,* and nerve growth factor inducible (*VGF*), that may be possibly related with side effects reported after finasteride treatment (Fig. 1B).

To investigate the transcriptional programs modulated by finasteride in hypothalamus at T0, we carried out Gene-Set Enrichment Analyses (GSEA) using the classical GSEA hallmarks as reference gene-sets. Using this approach we identified the hallmark WNT_BETA_CATENIN_SIGNALING as significantly enriched in finasteride-treated hypothalamus at T0 (Fig. 1 C,D; Normalized Enrichment Score (NES) 1.40; *p*_adj_ = 0.24). Differential expression analysis performed in the hippocampus at T1 failed to identify dysregulated genes (Supplementary Table 2), which suggests a modest transcriptional effect of finasteride at this timepoint. However, GSEA performed at T1 revealed a significant positive enrichment (Fig. 1E,F; NES 1.36; *p*_adj_ = 0.23) of the hallmark IL6_JAK_STAT3_SIGNALING.

**Fig. 2:**
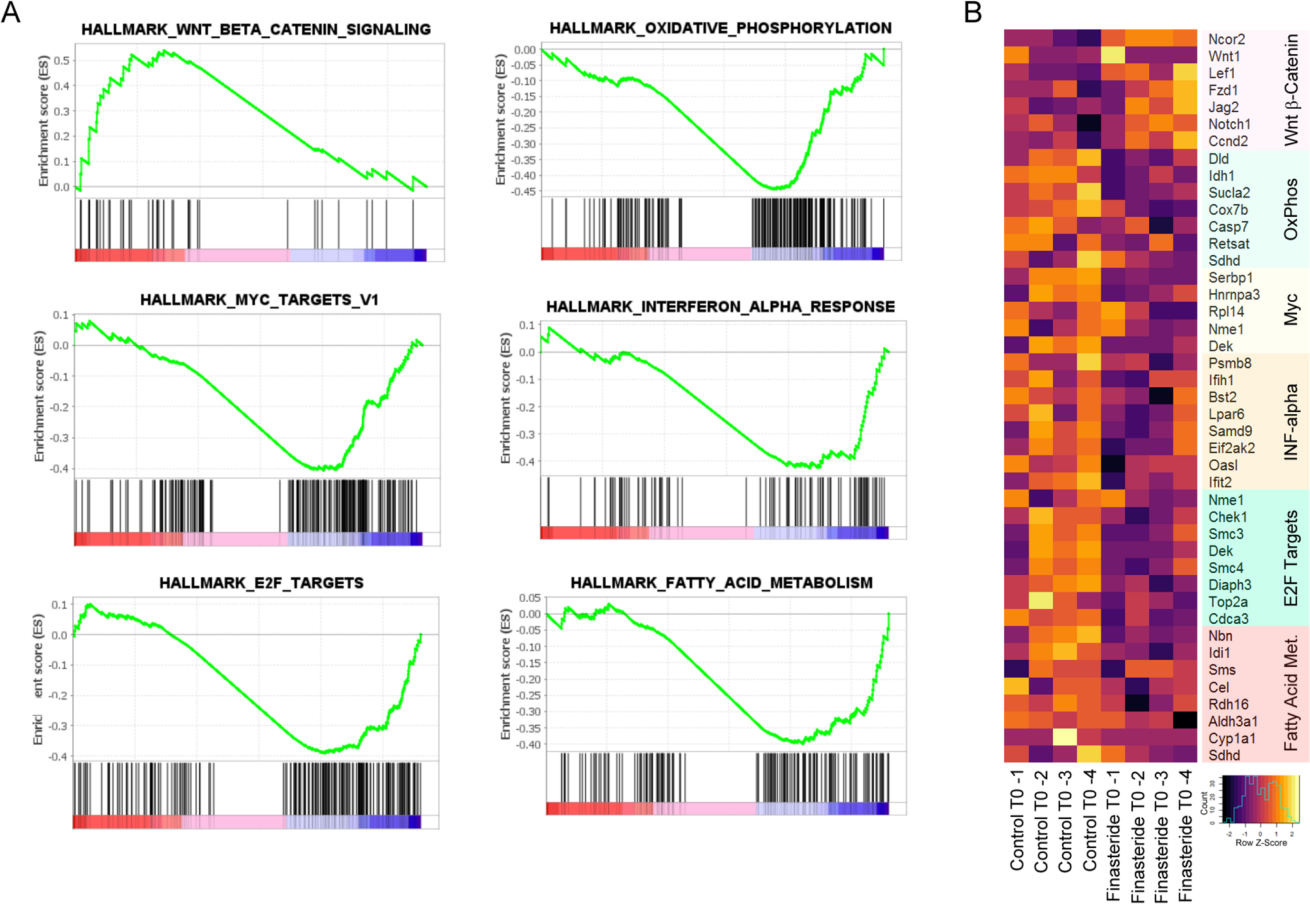
**A** GSEA plots showing positive and negative enrichment of specific gene-sets in Hippocampus at T0. **B** Heatmap reporting the leading genes associated with the GSEA shown in panel A sets in Hippocampus at T0 in control and Finasteride-treated rats. *n* = 4 for each experimental group

Data obtained in the hippocampus after chronic treatment with the drug showed that 19 genes were significantly affected, of them 17 were up and 2 downregulated (Supplementary Table 3). GSEA performed at T0 in the hippocampus revealed that, like in the case of hypothalamus (Fig. 1 C,D), the hallmark WNT_BETA_CATENIN_SIGNALING was significantly enriched (Fig. 2 A,B; NES 1.58; *p*_adj_ = 0.052). On the contrary, others hallmarks, such as OXIDATIVE_PHOSPHORYLATION (NES −1.59; *p*_adj_ = 0.037), MYC_TARGETS_V1 (NES −1.43; *p*_adj_ = 0.13), INTERFERON_ALPHA_RESPONSE (NES −1.37; *p*_adj_ = 0.088), E2F_TARGETS (NES −1.32; *p*_adj_ = 0.10), and FATTY_ACID_METABOLISM (NES −1.39; *p*_adj_ = 0.10) were significantly decreased (Fig. 2A,B).

**Fig. 3:**
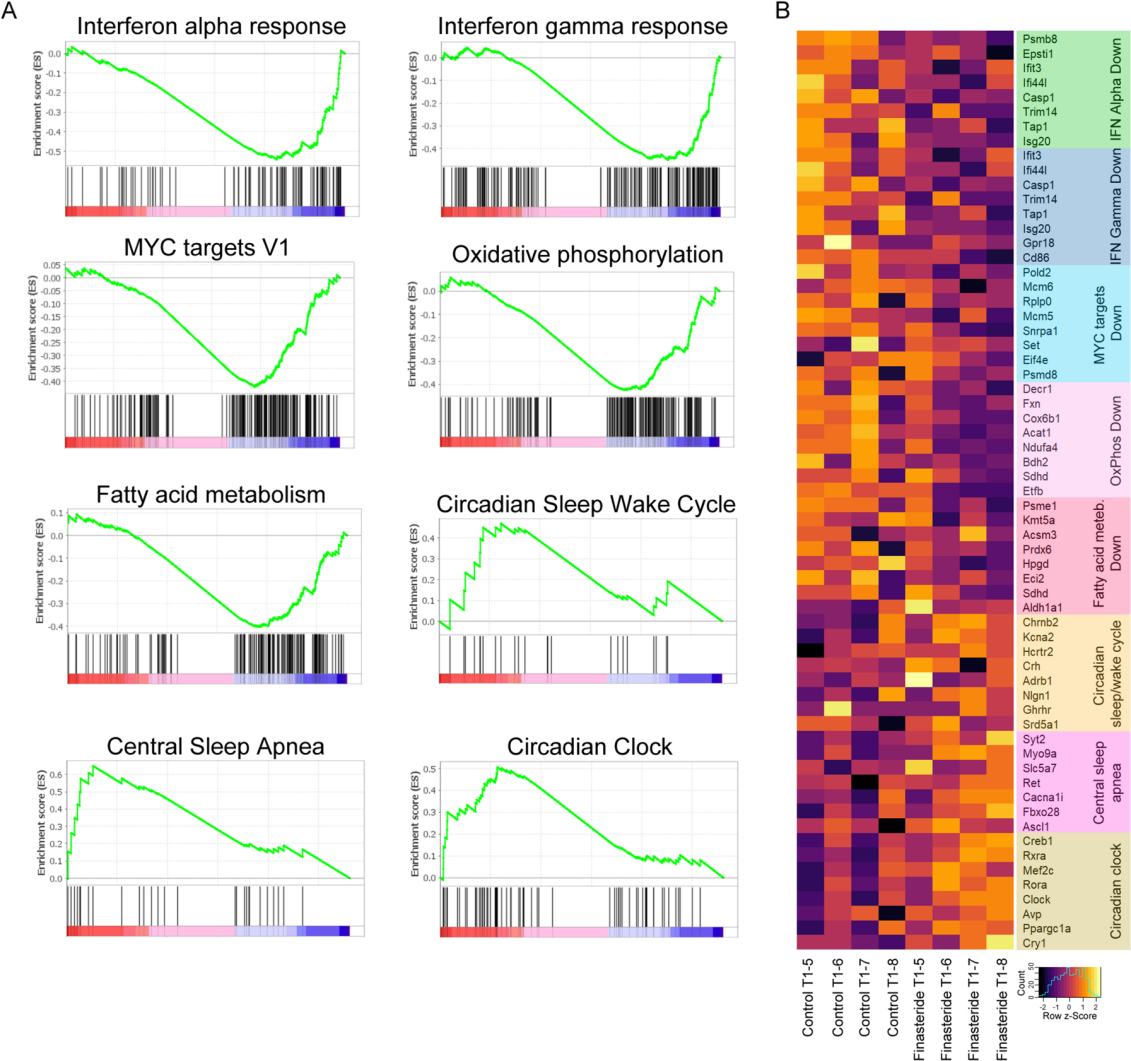
**A** GSEA plots showing positive and negative enrichment of specific gene-sets in Hippocampus at T1. **B** Heatmap reporting the leading genes associated with the GSEA shown in panel A sets in Hippocampus at T1 in control and Finasteride-treated rats. *n* = 4 for each experimental group

Differential expression analysis performed in the hippocampus  at T1 failed to identify dysregulated genes (Supplementary Table 4), however, GSEA revealed a decrease in the INTERFERON_ALPHA_RESPONSE (NES -1.73; p_adj_ = 0.005) and INTERFERON_GAMMA_RESPONSE hallmark (NES −1.57; p_adj_ = 0.028). Notably, MYC_TARGETS_V1 (NES −1.48; padj = 0.028), OXIDATIVE_PHOSPHORYLATION (NES −1.49; *p*_adj_ = 0.029) and FATTY_ACID_METABOLISM (NES −1.38; *p*_adj_ = 0.069) hallmarks were also downmodulated not only at T0 (Fig. 2A,B) but also at T1 (Fig. 3A,B). Interestingly, a significant enrichment of the WNT_BETA_CATENIN_SIGNALING hallmark present in this brain area at T0 (Fig. 1C,D) was still present at T1 (Supplementary Fig. 1; NES 1.43; *p*_adj_ = 0.11). In addition, an enrichment in hallmarks such as HP_CENTRAL_SLEEP_APNEA (Fig. 3A,B; NES 1.74; *p*_adj_ = 0.028), REACTOME_CIRCADIAN_CLOCK (Fig. 3A,B; NES 1.62; *p*_adj_ = 0.051) and GOBP_CIRCADIAN_SLEEP_WAKE_CYCLE (Fig. 3A,B; NES 1.22; *p*_adj_ = 0.23) was also observed.

## Discussion

Data here obtained by RNA sequencing showed that chronic treatment (i.e., for 20 days) with finasteride affects the expression of hypothalamic and hippocampal rat genes. As we reported, the most affected brain area is the hypothalamus, with 15 genes downregulated and 171 genes upregulated. Among the downregulated genes, we will here discuss those that, based on the literature available, could be associated with the side effects reported by the patients during the treatment and observed in the experimental model. For instance, *TTR* encodes for a carrier protein involved in the transport of thyroxine (T4) and retinol. Besides its role as a carrier protein, downregulation of this gene induces learning and memory impairment, aggressive behavior, and neurodegeneration [32–35]. In the context of the effects of thyroid hormones in the nervous system, it is important to highlight that we also observed a significant decrease in the gene *DIO2*. This gene encodes for the enzyme responsible for the conversion of prohormone T4 into the biologically active thyroid hormone, triiodothyronine (T3). Therefore, impairment in this enzymatic conversion may affect the important role exerted by T3 in brain functionality (e.g., on synaptic plasticity, oxidative stress, inflammation, mood, and neurotransmitter regulation) by genomic and nongenomic mechanisms [36–41]. Indeed, as demonstrated in adult mice lacking *DIO2*, reduced expression of several target genes of tyroid hormones [42, 43], altered motor ability [44], emotional alteration with increased anxiety-like behavior as well as enhanced fear memory was observed [45].

Other genes downregulated in the hypothalamus of rats chronically treated with finasteride are *CLDN2* and *CLDN1*. Claudin proteins are functional and structural components of tight junctions [46] that in the nervous system, apart from maintaining blood–brain barriers, also play important roles in maintaining the synaptic and neuronal structure and function. In line with these observations, alteration of these genes is related to neuropathological events [47]. Other genes downregulated are *SLC4A5* and *KCNE2*, also known to exert key roles in the nervous system. For instance, *SLC4A5* encodes Na+/HCO_3_- cotransporter 4, a membrane protein that plays a critical role in maintaining pH and ion balance in cells by transporting sodium and bicarbonate ions [48, 49]. Multiple defects were observed in the nervous system of *SLC4A5* deficient mice, such as decreased volume of lateral brain ventricles, decreased intracranial pressure, changes in the choroid plexus epithelium cell morphology and changes in cerebrospinal fluid composition [50]. Mice lacking *KCNE2* showed increased behavioral responsiveness to stress and seizure susceptibility [51]. *CROT* is also downregulated by finasteride treatment in the hypothalamus. The encoded protein converts 4,8-dimethylnonanoyl-CoA to its corresponding carnitine ester. This transesterification occurs in the peroxisome and is necessary for transport of medium- and long-chain acyl-CoA molecules out of the peroxisome to the cytosol and mitochondria [52]. Therefore, the protein plays a role in lipid metabolism and fatty acid beta-oxidation. As demonstrated, at least in a model of hepatic cells, knockdown of *CROT* has an important impact on fatty acid profile, with increase in the amount of medium chain saturated fatty acid and unsaturated C24 [52]. Therefore these data may suggest a role for this gene in regulating the peroxisomal oxidative pathway. In the brain, peroxisomes are mainly located in astrocytes and oligodendrocytes [53]. Dysfunction of peroxisomal mechanisms has been linked to alterations in the nervous system, such as demyelination, oxidative stress, and neuroinflammation [54].

Notably, upregulated genes were also identified upon treatment with finasteride. Among these, it is interesting to discuss *HCRT*. This gene encodes a hypothalamic neuropeptide precursor protein that gives rise to two mature neuropeptides, orexin A and orexin B. These two molecules play a significant role in the regulation of sleep-wakefulness [55]. Indeed, orexin system deficiency is associated with narcolepsy in animal models [56, 57] and in human [58–60]. Accordingly, treatment with orexin caused wakefulness and suppressed sleep in animal models [61–63]. In addition, alteration in orexin system is also associated with psychiatric disorders. For instance, hyperactivity of the system is related to acute stress reactions, depression, and anxiety-like behavior [55]. In this context, we also reported upregulation of myristoylated alanin-rich C-kinase (*MARCKSL1*). As demonstrated in transgenic mice, overexpression of this gene is associated with anxiety-like behavior [64]. In addition, other genes upregulated after finasteride treatment in the hypothalamus, like *VGF* and *IRF2BPL*, are associated with neurological disorders*.* The protein encoded by *VGF* is exclusively synthesized in neuronal and neuroendocrine cells [65, 66]. Mice overexpressing *VGF* showed behavioral abnormalities, such as hyperactivity, memory impairment, lower sociality, and higher depressive state, as well as morphological alterations, like smaller brain weight, expansion of the lateral ventricle, striatal morphological abnormalities [67]. Alterations in *IRF2BPL* levels has been associated with neurological phenotypes [68, 69] and with major depressive disorder [70]. Altogether, these data indicate that genes modulated by treatment with finasteride in the rat brain are potentially linked to some of the side effects observed in patients during the drug treatment. In particular, the closer relationship seem to be with psychiatric and neurological domains (i.e., depression, anxiety, disturbance in memory and attention, sleep disturbance). This is further confirmed by the GSEA we performed in the hypothalamus and hippocampus. As reported here, the WNT_BETA_CATENIN_SIGNALING hallmark is significantly enriched by the finasteride treatment in both brain areas considered. An increase in WNT/β-catenin signaling has been reported to be associated with disturbance in circadian rhythms and sleep [71]. Moreover, in the hippocampus, after finasteride treatment we also observed a significant decrease in GSEA hallmarks, such as the OXIDATIVE_PHOSPHORYLATION, MYC_TARGETS_V1, INTERFERON_ALPHA_RESPONSE, E2F_TARGETS, and FATTY_ACID_METABOLISM, suggesting mitochondrial dysfunction, oxidative stress, neuroinflammation and impairment in synaptic plasticity that are important features of neurodegeneration and mood disorders [72–75]. Interestingly, a decrease in the hallmarks OXIDATIVE_PHOSPHORYLATION, MYC_TARGETS_V1, INTERFERON_ALPHA_RESPONSE, and FATTY_ACID_METABOLISM was still present at finasteride withdrawal, suggesting persistence of the side effects induced by the drug. Dysregulated neuroinflammation, impaired synaptic plasticity, as well as altered microglial activation, may be also suggested by a decrease in the INTERFERON_GAMMA_RESPONSE hallmark that was observed in the hippocampus upon withdrawal of finasteride [76–79]. Interestingly, in this brain area we also reported an enrichment in HP_CENTRAL_SLEEP_APNEA, REACTOME_CIRCADIAN_CLOCK, and GOBP_CIRCADIAN_SLEEP_WAKE_CYCLE hallmarks further suggesting a dysregulation of gene networks involved in sleep and mood disorders, as well as in cognitive processes [80, 81].

In conclusion, the data obtained here suggest interesting gene targets that could be related to some of the side effects observed during finasteride treatment and withdrawal. Therefore, these data may provide an interesting background for future experiments addressed to confirm the pathological role of these genes in this experimental model, exploring the impact in their signaling pathways, and evaluating possible therapeutic strategy able to counteract their pathological effects.

## Supplementary Information

Below is the link to the electronic supplementary material.

Supplementary file1 (XLSX 3473 KB)

Supplementary file2 (XLSX 4134 KB)

Supplementary file3 (XLSX 3931 KB)

Supplementary file4 (XLSX 3952 KB)

Supplementary file5 (TIF 58 KB)

## Data Availability

Datasets generated during the current study are available from the corresponding author on reasonable request.

## References

[1] Kaufman KD, Olsen EA, Whiting D, Savin R, DeVillez R, Bergfeld W, Price VH, Van Neste D, Roberts JL, Hordinsky M, Shapiro J, Binkowitz B, Gormley GJ (1998) Finasteride in the treatment of men with androgenetic alopecia finasteride male pattern hair loss study group. J Am Acad Dermatol. 10.1016/S0190-9622(98)70007-6 9777765 10.1016/S0190-9622(98)70007-6

[2] Edwards JE, Moore RA (2002) Finasteride in the treatment of clinical benign prostatic hyperplasia: a systematic review of randomised trials. BMC Urol 2:14 12477383 10.1186/1471-2490-2-14 PMC140032

[3] Fwu CW, Eggers PW, Kirkali Z, McVary KT, Burrows PK, Kusek JW (2014) Change in sexual function in men with lower urinary tract symptoms/benign prostatic hyperplasia associated with long-term treatment with doxazosin, finasteride and combined therapy. J Urol 191(6):1828–1834. 10.1016/j.juro.2013.12.014 24342143 10.1016/j.juro.2013.12.014

[4] Traish AM, Melcangi RC, Bortolato M, Garcia-Segura LM, Zitzmann M (2015) Adverse effects of 5alpha-reductase inhibitors: What do we know, don’t know, and need to know? Rev Endocr Metab Disord 16:177–198. 10.1007/s11154-015-9319-y 26296373 10.1007/s11154-015-9319-y

[5] Belknap SM, Aslam I, Kiguradze T, Temps WH, Yarnold PR, Cashy J, Brannigan RE, Micali G, Nardone B, West DP (2015) Adverse event reporting in clinical trials of finasteride for androgenic alopecia: a meta-analysis. JAMA Dermatol 151(6):600–606. 10.1001/jamadermatol.2015.36 25830296 10.1001/jamadermatol.2015.36

[6] Diviccaro S, Melcangi RC, Giatti S (2020) Post-finasteride syndrome: an emerging clinical problem. Neurobiol Stress 12:100209. 10.1016/j.ynstr.2019.100209 32435662 10.1016/j.ynstr.2019.100209 PMC7231981

[7] Motofei IG, Rowland DL, Georgescu SR, Baconi DL, Dimcevici NP, Paunica S, Constantin VD, Balalau C (2013) A pilot study on the sexual side effects of finasteride as related to hand preference for men undergoing treatment of male pattern baldness. BJU Int. 10.1111/j.1464-410X.2012.11580.x 23157321 10.1111/j.1464-410X.2012.11580.x

[8] Motofei IG, Rowland DL, Georgescu SR, Tampa M, Baconi D, Stefanescu E, Baleanu BC, Balalau C, Constantin V, Paunica S (2016) Finasteride adverse effects in subjects with androgenic alopecia: a possible therapeutic approach according to the lateralization process of the brain. J Dermatol Treat. 10.3109/09546634.2016.1161155 10.3109/09546634.2016.1161155 27046152

[9] Irwig MS, Kolukula S (2011) Persistent sexual side effects of finasteride for male pattern hair loss. J Sex Med 8(6):1747–1753. 10.1111/j.1743-6109.2011.02255.x 21418145 10.1111/j.1743-6109.2011.02255.x

[10] Irwig MS (2012) Persistent sexual side effects of finasteride: could they be permanent? J Sex Med 9(11):2927–2932. 10.1111/j.1743-6109.2012.02846.x 22789024 10.1111/j.1743-6109.2012.02846.x

[11] Ganzer CA, Jacobs AR, Iqbal F (2015) Persistent sexual, emotional, and cognitive impairment post-finasteride: a survey of men reporting symptoms. Am J Mens Health 9(3):222–228. 10.1177/1557988314538445 24928450 10.1177/1557988314538445

[12] Basaria S, Jasuja R, Huang G, Wharton W, Pan H, Pencina K, Li Z, Travison TG, Bhawan J, Gonthier R, Labrie F, Dury AY, Serra C, Papazian A, O’Leary M, Amr S, Storer TW, Stern E, Bhasin S (2016) Characteristics of men who report persistent sexual symptoms after finasteride use for hair loss. J Clin Endocrinol Metab 101(12):4669–4680. 10.1210/jc.2016-2726 27662439 10.1210/jc.2016-2726 PMC5155688

[13] Melcangi RC, Santi D, Spezzano R, Grimoldi M, Tabacchi T, Fusco ML, Diviccaro S, Giatti S, Carra G, Caruso D, Simoni M, Cavaletti G (2017) Neuroactive steroid levels and psychiatric and andrological features in post-finasteride patients. J Steroid Biochem Mol Biol 171:229–235. 10.1016/j.jsbmb.2017.04.003 28408350 10.1016/j.jsbmb.2017.04.003

[14] Khera M, Than JK, Anaissie J, Antar A, Song W, Losso B, Pastuszak A, Kohn T, Mirabal JR (2020) Penile vascular abnormalities in young men with persistent side effects after finasteride use for the treatment of androgenic alopecia. Transl Androl Urol 9(3):1201–1209. 10.21037/tau.2020.03.21 32676403 10.21037/tau.2020.03.21 PMC7354335

[15] Melcangi RC, Caruso D, Abbiati F, Giatti S, Calabrese D, Piazza F, Cavaletti G (2013) Neuroactive steroid levels are modified in cerebrospinal fluid and plasma of post-finasteride patients showing persistent sexual side effects and anxious/depressive symptomatology. J Sex Med 10(10):2598–2603. 10.1111/jsm.12269 23890183 10.1111/jsm.12269

[16] Caruso D, Abbiati F, Giatti S, Romano S, Fusco L, Cavaletti G, Melcangi RC (2015) Patients treated for male pattern hair with finasteride show, after discontinuation of the drug, altered levels of neuroactive steroids in cerebrospinal fluid and plasma. J Steroid Biochem Mol Biol 146:74–79. 10.1016/j.jsbmb.2014.03.012 24717976 10.1016/j.jsbmb.2014.03.012

[17] Giatti S, Foglio B, Romano S, Pesaresi M, Panzica G, Garcia-Segura LM, Caruso D, Melcangi RC (2016) Effects of subchronic finasteride treatment and withdrawal on neuroactive steroid levels and their receptors in the male rat brain. Neuroendocrinology 103(6):746–757. 10.1159/000442982 26646518 10.1159/000442982

[18] Di Loreto C, La Marra F, Mazzon G, Belgrano E, Trombetta C, Cauci S (2014) Immunohistochemical evaluation of androgen receptor and nerve structure density in human prepuce from patients with persistent sexual side effects after finasteride use for androgenetic alopecia. PLoS ONE 9(6):e100237. 10.1371/journal.pone.0100237 24959691 10.1371/journal.pone.0100237 PMC4069023

[19] Cecchin E, De Mattia E, Mazzon G, Cauci S, Trombetta C, Toffoli G (2014) A pharmacogenetic survey of androgen receptor (CAG)n and (GGN)n polymorphisms in patients experiencing long term side effects after finasteride discontinuation. Int J Biol Markers 29(4):e310-316. 10.5301/jbm.5000095 24855036 10.5301/jbm.5000095

[20] Cauci S, Chiriaco G, Cecchin E, Toffoli G, Xodo S, Stinco G, Trombetta C (2017) Androgen receptor (AR) gene (CAG)n and (GGN)n length polymorphisms and symptoms in young males with long-lasting adverse effects after finasteride use against androgenic alopecia. Sex Med 5(1):e61–e71. 10.1016/j.esxm.2016.11.001 28024997 10.1016/j.esxm.2016.11.001 PMC5302381

[21] Melcangi RC, Giatti S, Garcia-Segura LM (2016) Levels and actions of neuroactive steroids in the nervous system under physiological and pathological conditions: sex-specific features. Neurosci Biobehav Rev 67:25–40. 10.1016/j.neubiorev.2015.09.023 26657814 10.1016/j.neubiorev.2015.09.023

[22] Borgo F, Macandog AD, Diviccaro S, Falvo E, Giatti S, Cavaletti G, Melcangi RC (2020) Alterations of gut microbiota composition in post-finasteride patients: a pilot study. J Endocrinol Invest 44:1263–1273. 10.1007/s40618-020-01424-0 32951160 10.1007/s40618-020-01424-0 PMC8124058

[23] Diviccaro S, Giatti S, Cioffi L, Falvo E, Herian M, Caruso D, Melcangi RC (2022) Gut Inflammation induced by finasteride withdrawal: therapeutic effect of allopregnanolone in adult male rats. Biomolecules 12(11):1567. 10.3390/biom12111567 36358917 10.3390/biom12111567 PMC9687671

[24] Diviccaro S, Giatti S, Borgo F, Barcella M, Borghi E, Trejo JL, Garcia-Segura LM, Melcangi RC (2019) Treatment of male rats with finasteride, an inhibitor of 5alpha-reductase enzyme, induces long-lasting effects on depressive-like behavior, hippocampal neurogenesis, neuroinflammation and gut microbiota composition. Psychoneuroendocrinology 99:206–215. 10.1016/j.psyneuen.2018.09.021 30265917 10.1016/j.psyneuen.2018.09.021

[25] Diviccaro S, Herian M, Cioffi L, Audano M, Mitro N, Caruso D, Giatti S, Melcangi RC (2023) Exploring rat corpus cavernosum alterations induced by finasteride treatment and withdrawal. Andrology. 10.1111/andr.13515 37621185 10.1111/andr.13515

[26] Giatti S, Di Domizio A, Diviccaro S, Falvo E, Caruso D, Contini A, Melcangi RC (2021) Three-dimensional proteome-wide scale screening for the 5-alpha reductase inhibitor finasteride: identification of a novel off-target. J Med Chem 64(8):4553–4566. 10.1021/acs.jmedchem.0c02039 33843213 10.1021/acs.jmedchem.0c02039 PMC8154553

[27] Howell S, Song W, Pastuszak A, Khera M (2021) Differential gene expression in post-finasteride syndrome patients. J Sex Med 18(9):1479–1490. 10.1016/j.jsxm.2021.05.009 34247957 10.1016/j.jsxm.2021.05.009

[28] Dobin A, Davis CA, Schlesinger F, Drenkow J, Zaleski C, Jha S, Batut P, Chaisson M, Gingeras TR (2013) STAR: ultrafast universal RNA-seq aligner. Bioinformatics 29(1):15–21. 10.1093/bioinformatics/bts635 23104886 10.1093/bioinformatics/bts635 PMC3530905

[29] Love MI, Huber W, Anders S (2014) Moderated estimation of fold change and dispersion for RNA-seq data with DESeq2. Genome Biol 15(12):550. 10.1186/s13059-014-0550-8 25516281 10.1186/s13059-014-0550-8 PMC4302049

[30] Danecek P, Bonfield JK, Liddle J, Marshall J, Ohan V, Pollard MO, Whitwham A, Keane T, McCarthy SA, Davies RM, Li H (2021) Twelve years of SAMtools and BCFtools. Gigascience. 10.1093/gigascience/giab008 33590861 10.1093/gigascience/giab008 PMC7931819

[31] Thorvaldsdottir H, Robinson JT, Mesirov JP (2013) Integrative Genomics Viewer (IGV): high-performance genomics data visualization and exploration. Brief Bioinform 14(2):178–192. 10.1093/bib/bbs017 22517427 10.1093/bib/bbs017 PMC3603213

[32] Fleming CE, Mar FM, Franquinho F, Saraiva MJ, Sousa MM (2009) Transthyretin internalization by sensory neurons is megalin mediated and necessary for its neuritogenic activity. J Neurosci 29(10):3220–3232. 10.1523/JNEUROSCI.6012-08.2009 19279259 10.1523/JNEUROSCI.6012-08.2009 PMC6666452

[33] Doggui S, Brouillette J, Chabot JG, Farso M, Quirion R (2010) Possible involvement of transthyretin in hippocampal beta-amyloid burden and learning behaviors in a mouse model of alzheimer’s disease (TgCRND8). Neurodegener Dis 7(1–3):88–95. 10.1159/000285513 20173334 10.1159/000285513

[34] Nunes AF, Montero M, Franquinho F, Santos SD, Malva J, Zimmer J, Sousa MM (2009) Transthyretin knockout mice display decreased susceptibility to AMPA-induced neurodegeneration. Neurochem Int 55(7):454–457. 10.1016/j.neuint.2009.07.001 19595729 10.1016/j.neuint.2009.07.001

[35] Sousa JC, Marques F, Dias-Ferreira E, Cerqueira JJ, Sousa N, Palha JA (2007) Transthyretin influences spatial reference memory. Neurobiol Learn Mem 88(3):381–385. 10.1016/j.nlm.2007.07.006 17698379 10.1016/j.nlm.2007.07.006

[36] Fernandez-Lamo I, Montero-Pedrazuela A, Delgado-Garcia JM, Guadano-Ferraz A, Gruart A (2009) Effects of thyroid hormone replacement on associative learning and hippocampal synaptic plasticity in adult hypothyroid rats. Eur J Neurosci 30(4):679–692. 10.1111/j.1460-9568.2009.06862.x 19686470 10.1111/j.1460-9568.2009.06862.x

[37] Chang H, Lin C, Li Z, Shen Y, Zhang G, Mao L, Ma C, Liu N, Lu H (2022) T3 alleviates neuroinflammation and reduces early brain injury after subarachnoid haemorrhage by promoting mitophagy via PINK 1-parkin pathway. Exp Neurol 357:114175. 10.1016/j.expneurol.2022.114175 35868360 10.1016/j.expneurol.2022.114175

[38] Bauer M, Heinz A, Whybrow PC (2002) Thyroid hormones, serotonin and mood: of synergy and significance in the adult brain. Mol Psychiatry 7(2):140–156. 10.1038/sj.mp.4000963 11840307 10.1038/sj.mp.4000963

[39] Joffe RT, Sokolov ST (1994) Thyroid hormones, the brain, and affective disorders. Crit Rev Neurobiol 8(1–2):45–63 8124730

[40] Chakrabarti N, Sarkar PK, Ray AK, Martin JV (2023) Unveiling the nongenomic actions of thyroid hormones in adult mammalian brain: the legacy of Mary B Dratman. Front Endocrinol (Lausanne) 14:1240265. 10.3389/fendo.2023.1240265 37842308 10.3389/fendo.2023.1240265 PMC10570802

[41] Murolo M, Di Vincenzo O, Cicatiello AG, Scalfi L, Dentice M (2022) Cardiovascular and neuronal consequences of thyroid hormones alterations in the ischemic stroke. Metabolites. 10.3390/metabo13010022 36676947 10.3390/metabo13010022 PMC9863748

[42] Galton VA, Wood ET, St Germain EA, Withrow CA, Aldrich G, St Germain GM, Clark AS, St Germain DL (2007) Thyroid hormone homeostasis and action in the type 2 deiodinase-deficient rodent brain during development. Endocrinology 148(7):3080–3088. 10.1210/en.2006-1727 17332058 10.1210/en.2006-1727

[43] Galton VA, Schneider MJ, Clark AS, St Germain DL (2009) Life without thyroxine to 3,5,3′-triiodothyronine conversion: studies in mice devoid of the 5′-deiodinases. Endocrinology 150(6):2957–2963. 10.1210/en.2008-1572 19196796 10.1210/en.2008-1572 PMC2689801

[44] Barez-Lopez S, Bosch-Garcia D, Gomez-Andres D, Pulido-Valdeolivas I, Montero-Pedrazuela A, Obregon MJ, Guadano-Ferraz A (2014) Abnormal motor phenotype at adult stages in mice lacking type 2 deiodinase. PLoS ONE 9(8):e103857. 10.1371/journal.pone.0103857 25083788 10.1371/journal.pone.0103857 PMC4118963

[45] Barez-Lopez S, Montero-Pedrazuela A, Bosch-Garcia D, Venero C, Guadano-Ferraz A (2017) Increased anxiety and fear memory in adult mice lacking type 2 deiodinase. Psychoneuroendocrinology 84:51–60. 10.1016/j.psyneuen.2017.06.013 28654773 10.1016/j.psyneuen.2017.06.013

[46] Schneeberger EE, Lynch RD (2004) The tight junction: a multifunctional complex. Am J Physiol Cell Physiol 286(6):C1213-1228. 10.1152/ajpcell.00558.2003 15151915 10.1152/ajpcell.00558.2003

[47] Tikiyani V, Babu K (2019) Claudins in the brain: unconventional functions in neurons. Traffic 20(11):807–814. 10.1111/tra.12685 31418988 10.1111/tra.12685

[48] Damkier HH, Nielsen S, Praetorius J (2007) Molecular expression of SLC4-derived Na+-dependent anion transporters in selected human tissues. Am J Physiol Regul Integr Comp Physiol 293(5):R2136-2146. 10.1152/ajpregu.00356.2007 17715183 10.1152/ajpregu.00356.2007

[49] Christensen HL, Nguyen AT, Pedersen FD, Damkier HH (2013) Na(+) dependent acid-base transporters in the choroid plexus; insights from slc4 and slc9 gene deletion studies. Front Physiol 4:304. 10.3389/fphys.2013.00304 24155723 10.3389/fphys.2013.00304 PMC3804831

[50] Kao L, Kurtz LM, Shao X, Papadopoulos MC, Liu L, Bok D, Nusinowitz S, Chen B, Stella SL, Andre M, Weinreb J, Luong SS, Piri N, Kwong JM, Newman D, Kurtz I (2011) Severe neurologic impairment in mice with targeted disruption of the electrogenic sodium bicarbonate cotransporter NBCe2 (Slc4a5 gene). J Biol Chem 286(37):32563–32574. 10.1074/jbc.M111.249961 21705333 10.1074/jbc.M111.249961 PMC3173174

[51] Abbott GW, Tai KK, Neverisky DL, Hansler A, Hu Z, Roepke TK, Lerner DJ, Chen Q, Liu L, Zupan B, Toth M, Haynes R, Huang X, Demirbas D, Buccafusca R, Gross SS, Kanda VA, Berry GT (2014) KCNQ1, KCNE2, and Na+-coupled solute transporters form reciprocally regulating complexes that affect neuronal excitability. Sci Signal. 10.1126/scisignal.2005025 24595108 10.1126/scisignal.2005025 PMC4063528

[52] Le Borgne F, Ben Mohamed A, Logerot M, Garnier E, Demarquoy J (2011) Changes in carnitine octanoyltransferase activity induce alteration in fatty acid metabolism. Biochem Biophys Res Commun 409(4):699–704. 10.1016/j.bbrc.2011.05.068 21619872 10.1016/j.bbrc.2011.05.068

[53] Rose J, Brian C, Pappa A, Panayiotidis MI, Franco R (2020) Mitochondrial metabolism in astrocytes regulates brain bioenergetics. Neurotransmission Redox Balance Front Neurosci 14:536682. 10.3389/fnins.2020.536682 33224019 10.3389/fnins.2020.536682 PMC7674659

[54] Trompier D, Vejux A, Zarrouk A, Gondcaille C, Geillon F, Nury T, Savary S, Lizard G (2014) Brain peroxisomes. Biochimie 98:102–110. 10.1016/j.biochi.2013.09.009 24060512 10.1016/j.biochi.2013.09.009

[55] Ten-Blanco M, Flores A, Cristino L, Pereda-Perez I, Berrendero F (2023) Targeting the orexin/hypocretin system for the treatment of neuropsychiatric and neurodegenerative diseases: from animal to clinical studies. Front Neuroendocrinol 69:101066. 10.1016/j.yfrne.2023.101066 37015302 10.1016/j.yfrne.2023.101066

[56] Chemelli RM, Willie JT, Sinton CM, Elmquist JK, Scammell T, Lee C, Richardson JA, Williams SC, Xiong Y, Kisanuki Y, Fitch TE, Nakazato M, Hammer RE, Saper CB, Yanagisawa M (1999) Narcolepsy in orexin knockout mice: molecular genetics of sleep regulation. Cell 98(4):437–451. 10.1016/s0092-8674(00)81973-x 10481909 10.1016/s0092-8674(00)81973-x

[57] Lin L, Faraco J, Li R, Kadotani H, Rogers W, Lin X, Qiu X, de Jong PJ, Nishino S, Mignot E (1999) The sleep disorder canine narcolepsy is caused by a mutation in the hypocretin (orexin) receptor 2 gene. Cell 98(3):365–376. 10.1016/s0092-8674(00)81965-0 10458611 10.1016/s0092-8674(00)81965-0

[58] Peyron C, Faraco J, Rogers W, Ripley B, Overeem S, Charnay Y, Nevsimalova S, Aldrich M, Reynolds D, Albin R, Li R, Hungs M, Pedrazzoli M, Padigaru M, Kucherlapati M, Fan J, Maki R, Lammers GJ, Bouras C, Kucherlapati R, Nishino S, Mignot E (2000) A mutation in a case of early onset narcolepsy and a generalized absence of hypocretin peptides in human narcoleptic brains. Nat Med 6(9):991–997. 10.1038/79690 10973318 10.1038/79690

[59] Thannickal TC, Moore RY, Nienhuis R, Ramanathan L, Gulyani S, Aldrich M, Cornford M, Siegel JM (2000) Reduced number of hypocretin neurons in human narcolepsy. Neuron 27(3):469–474. 10.1016/s0896-6273(00)00058-1 11055430 10.1016/s0896-6273(00)00058-1 PMC8760623

[60] Nishino S, Ripley B, Overeem S, Lammers GJ, Mignot E (2000) Hypocretin (orexin) deficiency in human narcolepsy. Lancet 355(9197):39–40. 10.1016/S0140-6736(99)05582-8 10615891 10.1016/S0140-6736(99)05582-8

[61] Mieda M, Willie JT, Hara J, Sinton CM, Sakurai T, Yanagisawa M (2004) Orexin peptides prevent cataplexy and improve wakefulness in an orexin neuron-ablated model of narcolepsy in mice. Proc Natl Acad Sci U S A 101(13):4649–4654. 10.1073/pnas.0400590101 15070772 10.1073/pnas.0400590101 PMC384801

[62] Mieda M, Hasegawa E, Kisanuki YY, Sinton CM, Yanagisawa M, Sakurai T (2011) Differential roles of orexin receptor-1 and -2 in the regulation of non-REM and REM sleep. J Neurosci 31(17):6518–6526. 10.1523/JNEUROSCI.6506-10.2011 21525292 10.1523/JNEUROSCI.6506-10.2011 PMC3732784

[63] Thakkar MM, Ramesh V, Strecker RE, McCarley RW (2001) Microdialysis perfusion of orexin-A in the basal forebrain increases wakefulness in freely behaving rats. Arch Ital Biol 139(3):313–328 11330208

[64] Tanaka T, Shimizu S, Ueno M, Fujihara Y, Ikawa M, Miyata S (2018) MARCKSL1 regulates spine formation in the amygdala and controls the hypothalamic-pituitary-adrenal axis and anxiety-like behaviors. EBioMedicine 30:62–73. 10.1016/j.ebiom.2018.03.018 29580842 10.1016/j.ebiom.2018.03.018 PMC5952351

[65] Snyder SE, Salton SR (1998) Expression of VGF mRNA in the adult rat central nervous system. J Comp Neurol 394(1):91–105 9550144 10.1002/(SICI)1096-9861(19980427)394:1<91::AID-CNE7>3.0.CO;2-C

[66] van den Pol AN, Bina K, Decavel C, Ghosh P (1994) VGF expression in the brain. J Comp Neurol 347(3):455–469. 10.1002/cne.903470311 7822494 10.1002/cne.903470311

[67] Mizoguchi T, Minakuchi H, Ishisaka M, Tsuruma K, Shimazawa M, Hara H (2017) Behavioral abnormalities with disruption of brain structure in mice overexpressing VGF. Sci Rep 7(1):4691. 10.1038/s41598-017-04132-7 28680036 10.1038/s41598-017-04132-7 PMC5498671

[68] Marcogliese PC, Shashi V, Spillmann RC, Stong N, Rosenfeld JA, Koenig MK, Martinez-Agosto JA, Herzog M, Chen AH, Dickson PI, Lin HJ, Vera MU, Salamon N, Graham JM Jr, Ortiz D, Infante E, Steyaert W, Dermaut B, Poppe B, Chung HL, Zuo Z, Lee PT, Kanca O, Xia F, Yang Y, Smith EC, Jasien J, Kansagra S, Spiridigliozzi G, El-Dairi M, Lark R, Riley K, Koeberl DD, Golden-Grant K, Program for Undiagnosed D, Undiagnosed Diseases N, Yamamoto S, Wangler MF, Mirzaa G, Hemelsoet D, Lee B, Nelson SF, Goldstein DB, Bellen HJ, Pena LDM, (2018) IRF2BPL is associated with Neurological phenotypes. Am J Hum Genet 103(3):456. 10.1016/j.ajhg.2018.08.010 30193138 10.1016/j.ajhg.2018.08.010 PMC6128320

[69] Marcogliese PC, Shashi V, Spillmann RC, Stong N, Rosenfeld JA, Koenig MK, Martinez-Agosto JA, Herzog M, Chen AH, Dickson PI, Lin HJ, Vera MU, Salamon N, Graham JM Jr, Ortiz D, Infante E, Steyaert W, Dermaut B, Poppe B, Chung HL, Zuo Z, Lee PT, Kanca O, Xia F, Yang Y, Smith EC, Jasien J, Kansagra S, Spiridigliozzi G, El-Dairi M, Lark R, Riley K, Koeberl DD, Golden-Grant K, Program for Undiagnosed D, Undiagnosed Diseases N, Yamamoto S, Wangler MF, Mirzaa G, Hemelsoet D, Lee B, Nelson SF, Goldstein DB, Bellen HJ, Pena LDM, (2018) IRF2BPL Is Associated with Neurological phenotypes. Am J Hum Genet 103(2):245–260. 10.1016/j.ajhg.2018.07.006 30057031 10.1016/j.ajhg.2018.07.006 PMC6081494

[70] Li YJ, Kresock E, Kuplicki R, Savitz J, McKinney BA (2022) Differential expression of MDGA1 in major depressive disorder. Brain Behav Immun Health 26:100534. 10.1016/j.bbih.2022.100534 36247836 10.1016/j.bbih.2022.100534 PMC9563614

[71] Vallee A, Lecarpentier Y, Guillevin R, Vallee JN (2020) The influence of circadian rhythms and aerobic glycolysis in autism spectrum disorder. Transl Psychiatry 10(1):400. 10.1038/s41398-020-01086-9 33199680 10.1038/s41398-020-01086-9 PMC7669888

[72] Lin MT, Beal MF (2006) Mitochondrial dysfunction and oxidative stress in neurodegenerative diseases. Nature 443(7113):787–795. 10.1038/nature05292 17051205 10.1038/nature05292

[73] Felger JC, Li Z, Haroon E, Woolwine BJ, Jung MY, Hu X, Miller AH (2016) Inflammation is associated with decreased functional connectivity within corticostriatal reward circuitry in depression. Mol Psychiatry 21(10):1358–1365. 10.1038/mp.2015.168 26552591 10.1038/mp.2015.168 PMC4862934

[74] Reddy PH (2009) Role of mitochondria in neurodegenerative diseases: mitochondria as a therapeutic target in Alzheimer’s disease. CNS Spectr. 10.1017/s1092852900024901 19890241 10.1017/s1092852900024901 PMC3056539

[75] Salminen A, Ojala J, Kaarniranta K, Haapasalo A, Hiltunen M, Soininen H (2011) Astrocytes in the aging brain express characteristics of senescence-associated secretory phenotype. Eur J Neurosci 34(1):3–11. 10.1111/j.1460-9568.2011.07738.x 21649759 10.1111/j.1460-9568.2011.07738.x

[76] Li Q, Barres BA (2018) Microglia and macrophages in brain homeostasis and disease. Nat Rev Immunol 18(4):225–242. 10.1038/nri.2017.125 29151590 10.1038/nri.2017.125

[77] Hong S, Beja-Glasser VF, Nfonoyim BM, Frouin A, Li S, Ramakrishnan S, Merry KM, Shi Q, Rosenthal A, Barres BA, Lemere CA, Selkoe DJ, Stevens B (2016) Complement and microglia mediate early synapse loss in alzheimer mouse models. Science 352(6286):712–716. 10.1126/science.aad8373 27033548 10.1126/science.aad8373 PMC5094372

[78] Boulanger LM (2009) Immune proteins in brain development and synaptic plasticity. Neuron 64(1):93–109. 10.1016/j.neuron.2009.09.001 19840552 10.1016/j.neuron.2009.09.001

[79] Perry VH, Nicoll JA, Holmes C (2010) Microglia in neurodegenerative disease. Nat Rev Neurol 6(4):193–201. 10.1038/nrneurol.2010.17 20234358 10.1038/nrneurol.2010.17

[80] McClung CA (2007) Circadian genes, rhythms and the biology of mood disorders. Pharmacol Ther 114(2):222–232. 10.1016/j.pharmthera.2007.02.003 17395264 10.1016/j.pharmthera.2007.02.003 PMC1925042

[81] Morin CM, Benca R (2012) Chronic insomnia. Lancet 379(9821):1129–1141. 10.1016/S0140-6736(11)60750-2 22265700 10.1016/S0140-6736(11)60750-2

