# Supplementary figures and images for: Analysis of the finasteride treatment and its withdrawal in the rat hypothalamus and hippocampus at whole-transcriptome level
Source: J Endocrinol Invest. 2024 Mar 17;47(10):2565–74. doi: 10.1007/s40618-024-02345-y (PMC11393021; doi:10.1007/s40618-024-02345-y)

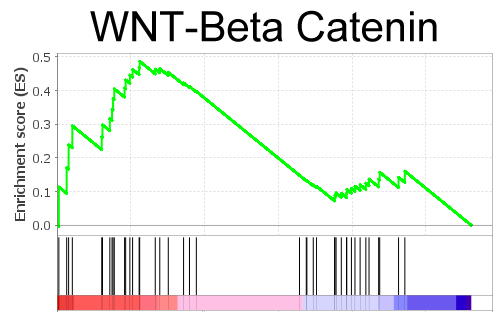

Supplement: Supplementary file 5 — Supplementary file5 (TIF 58 KB) [file 40618_2024_2345_MOESM5_ESM.tif]
